# Epidemiology, Clinical Features, and Prescribing Patterns of Irritable Bowel Syndrome in Taiwan

**DOI:** 10.3389/fphar.2021.788795

**Published:** 2021-12-09

**Authors:** Yu-Tung Lai, Chung-Yu Chen, Ming-Jong Bair

**Affiliations:** ^1^ Master Program in Clinical Pharmacy, School of Pharmacy, Kaohsiung Medical University, Kaohsiung, Taiwan; ^2^ Department of Pharmacy, Kaohsiung Medical University Hospital, Kaohsiung, Taiwan; ^3^ Department of Medical Research, Kaohsiung Medical University Hospital, Kaohsiung, Taiwan; ^4^ Division of Gastroenterology, Department of Internal Medicine, Taitung Mackay Memorial Hospital, Taitung, Taiwan; ^5^ Mackay Medical College, New Taipei, Taiwan

**Keywords:** epidemiology, irritable bowel syndrome, pharmacoepidaemiology, pattern, clinical features

## Abstract

**Background:** Understanding the prescribing patterns could better inform irritable bowel syndrome (IBS) management and health policy. However, there is no study on prescribing patterns of IBS in Taiwan. This study was conducted to evaluate the epidemiology, clinical features, and prescribing patterns of IBS in Taiwan.

**Methods:** This population-based cross-sectional study was performed by retrieving claim data from National Health Insurance Research Database (NHIRD) between 2011 and 2018 in Taiwan. Patients who were diagnosed with IBS during 2012–2018 and more than 20 years old were included. The annual incidence and prevalence of IBS were estimated. The characteristics and prescribing pattern were evaluated among IBS population. The population with IBS were followed from index date until 1 year after or death.

**Results:** A total of 1691596 patients diagnosed with IBS were identified from 2012 to 2018. The average annual incidence and prevalence of IBS in Taiwan were calculated as 106.54 and 181.75 per 10,000 population. The incidence and prevalence showed a decreasing trend from 2012 to 2018. Hypertension, dyslipidemia, chronic liver disease, peptic ulcer, gastroesophageal reflux disease (GERD), anxiety, and sleep disorder were the prevalent comorbidities in IBS population. At 1 year after IBS diagnosis, the rates of peptic ulcer and GERD; the utilizations of abdominal ultrasonography, upper gastrointestinal (GI) endoscopy, and lower GI endoscopy; the prescribing rate of propulsives, simethicone, antacids, H2-blockers, and proton pump inhibitors significantly increased. Approximately 70% of participants received IBS-related treatment. Antispasmodics was the most frequently prescribed medication class, followed by laxatives and antidiarrheals. Only 48.58% of patients made return visit for IBS at 1 year after IBS diagnosis. Consequently, the proportion of consultation for IBS and the prescribing rates of all medications were decreased considerably after IBS diagnosis.

**Conclusion:** The incidence and prevalence of IBS showed a decreasing trend from 2012 to 2018. More than two-third of patients received treatment for IBS. Antispasmodics was widely used for IBS management. However, patients may have a short symptom duration or receive a short course of IBS-related treatment in Taiwan. These findings provided the whole picture of the epidemiology and prescribing pattern of the IBS population in Taiwan.

## Introduction

Irritable bowel syndrome (IBS) is a common chronic functional disorder of the gastrointestinal (GI) tract characterized by chronic abdominal pain and altered bowel habits without organic disease (Masuy et al., 2020). Although IBS does not increase mortality risk, it impairs the quality of life and imposes a substantial economic burden on patients and healthcare systems (Black and Ford, 2020). The global prevalence of IBS in 2012 was estimated to be 11.2% (Lovell and Ford, 2012). Nevertheless, the prevalence varies considerably in each country. The variation might be attributed to racial, ethnic, and methodological variations between studies. In Taiwan, the prevalence of IBS ranged from 4.4 to 23.3% (Lu et al., 2003; Chang et al., 2012; Chang et al., 2015) and the overall incidence of IBS was estimated to be 51.27 per 10000 person-years (Pan et al., 2016). Despite the higher prevalence rate, the pathophysiology of IBS is still incompletely understood. Thus, the clinical practice for managing IBS targets individual symptom relief. However, it is difficult to choose the proper pharmacological treatment due to limited evidence-based treatment options. Most treatment choices depend on historical practices (Gwee et al., 2019; Moayyedi et al., 2019; Masuy et al., 2020; Fukudo et al., 2021; Lacy et al., 2021). Furthermore, the extensive use of non-pharmacological therapies and the habit of rotating treatments when one stops working must be considered (Bonetto et al., 2021). Unfortunately, no consensus guidelines on IBS management and literature on IBS prescribing patterns has been published in Taiwan. Additionally, some efficacious drugs approved for IBS are unavailable in Taiwan. Therefore, evaluating the prescribing patterns and appropriateness of medication in patients with IBS is crucial. Considering the lack of related information, we performed a nationwide population-based cross-sectional study to investigate the epidemiology and evaluate the prescribing pattern in patients with IBS in Taiwan.

## Methods

### Data Sources

We performed a population-based study using data from the National Health Insurance Research Database (NHIRD) from 2011 to 2018 in Taiwan. NHIRD contains registration files and medical benefit claim data, including demographics, clinical diagnoses, hospital discharge diagnoses, diagnostic tests and procedures, and prescriptions, for approximately 99.9% of people in Taiwan (Hsieh et al., 2019). The diagnoses are coded using the International Classification of Diseases, Ninth Edition, Clinical Modification (ICD-9-CM) and the Tenth Edition (ICD-10-CM) codes (Ellis et al., 2020). The prescriptions include generic names, brand names, dosage, durations, and the Anatomical Therapeutic Chemical (ATC) code. This study was approved by the Institutional Review Board (IRB) of Kaohsiung Medical University Chung-Ho Memorial Hospital (KMUHIRB-E(II)-20190359). The personal data, such as the names of patients, was encrypted with unique and anonymous identifiers. Thus, The IRB waived the requirement for consent.

### Study Population

We included patients diagnosed with IBS between 2012 and 2018 (ICD-9-CM code: 564.1 and ICD-10-CM code: K58.0 and K58.9). Patients with IBS were identified as those who had at least one outpatient or one inpatient diagnosis. Patients who were younger than 20 years old or whose age or gender data was missing were excluded for the evaluation of epidemiology. Subsequently, patients diagnosed in 2011 and newly diagnosed in 2018 were excluded for assessing clinical features and prescribing patterns of IBS. The index date is defined as the date of the first diagnosis of IBS.

### Baseline Characteristics

We collected information on patients’ demographics, diagnostic procedures, comorbidities, and comedications. Demographics including age, gender, and urbanization were extracted on the index date. The diagnostic procedure was defined as patients having at least one diagnostic procedure identified by the National Health Insurance (NHI) order code (Supplementary eTable S1). The diagnostic procedures included upper GI endoscopy, enteroscopy, lower GI endoscopy, abdominal X-ray, and ultrasonography. Comorbidity was defined as those who had at least two ambulatory or outpatient or one inpatient diagnosis (Supplementary eTable S2). The comorbidities were categorized into gastrointestinal, non-gastrointestinal, and psychiatric disorders. The GI diseases included gastritis and duodenitis, gastroenteritis and colitis, and gastroesophageal reflux disease (GERD). Non-GI diseases included hypertension, diabetes, dyslipidemia. The psychiatric disorders included depression, anxiety, and sleep disorders. The comedication user was defined as exposure over 14 days in each period. The comedications included simethicone, propulsives, antacids, H2-blockers, proton pump inhibitors (PPI), urinary antispasmodics, and anxiolytics (Supplementary eTable S3).

### Prescribing Pattern

We investigated the prescriptions for the treatment of IBS included laxatives, antidiarrheals, antispasmodics, antidepressants, and antibiotics. User was defined as patients who had one claim for IBS medication with an IBS diagnosis (Supplementary eTable S4). The population with IBS were followed from index date until 1 year after or death.

### Statistical Analysis

The crude annual incidence and prevalence of IBS from 2012 to 2018 were calculated. The 2012 population was used as the standard population for standardized incidence and prevalence calculation. The annual incidence and prevalence were estimated by dividing the number of incident and prevalent cases by the total Taiwanese population each year. Multivariate Poisson regression was used to analyze the trends and the effects of age and sex in incidence and prevalence with adjustment of insurance premium and urbanization level. Descriptive statistics were presented as means ± SD for the continuous variables and numbers and percentages for the categorical variables. Standardized mean difference (SMD) was used to assess the differences between years. The Cochran-Armitage test was conducted to analyze the prescription trend from index date until 1 year after.

## Results

A total of 1691596 patients diagnosed with IBS were identified in the NHIRD between 2012 and 2018 for the evaluation of epidemiology. After excluding patients diagnosed in 2011 and newly diagnosed in 2018, 1193490 patients were included in the subsequent analyses (Supplementary eFigure S1).

### Epidemiology of IBS

**FIGURE 1:**
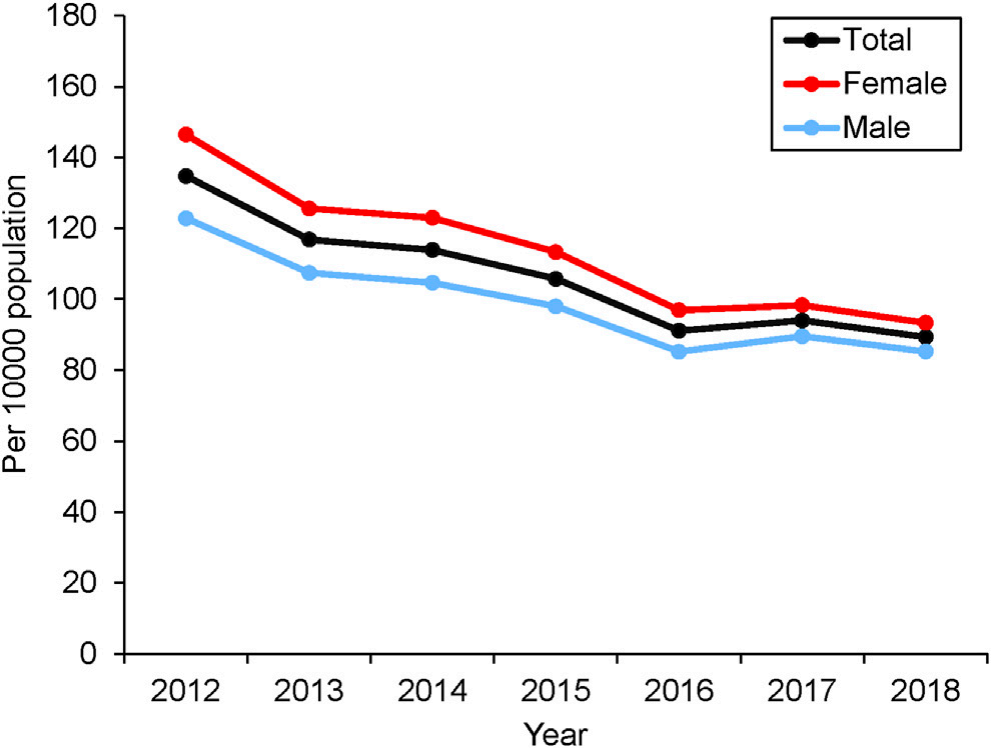
The annual incidence of IBS during 2012–2018.

The overall incidence of IBS was 106.54 per 10000 population. The crude annual incidence of IBS showed a declining trend from 134.79 to 89.35 per 10000 population with a decrease of 33.71% from 2012 to 2018 (Figure 1). The standardized annual incidence decreased 34.34% from 134.79 in 2012 to 88.37 per 10000 population in 2018. The annual incidence in both genders decreased over time. The incidence for females was higher than males (Supplementary eTable S5). Multivariate Poisson regression indicated that the calendar year, age, and female gender are significantly associated with the incidence of IBS (Supplementary eTable S6). It showed significant declines in the adjusted incidence rate ratio (IRR) of IBS from 2012 to 2018 (0.91, [95% CI 0.91-0.91]; *p* < 0.001). However, the incidence of IBS increased with increasing age (1.01, [95% CI 1.01-1.01]; *p* < 0.001) and was predominant in females (1.14 [95% CI 1.14-1.15]; *p* < 0.001).

**FIGURE 2:**
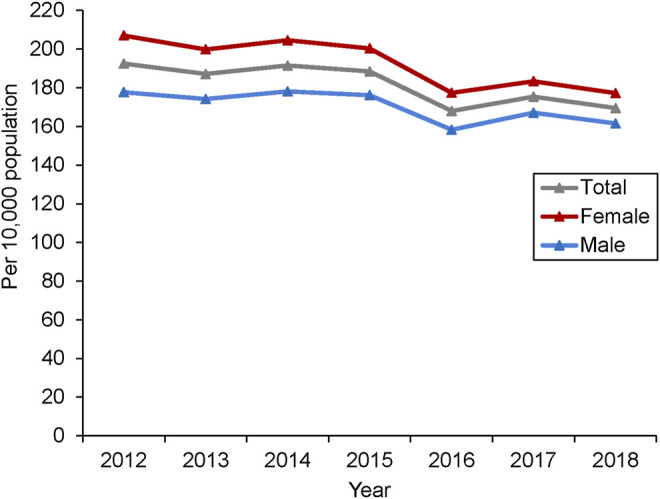
The annual prevalence of IBS during 2012–2018.

The overall prevalence of IBS was 181.75 per 10000 population. The crude annual prevalence of IBS fluctuated between 2012 and 2015 and decreased sharply in 2016. After that, it decreased to 169.47 per 10,000 population in 2018 (Figure 2). Overall, the prevalence decreased 11.94% from 2012 to 2018. The age-standardized annual prevalence was slightly lower than the crude annual prevalence, which decreased 13.55% from 2012 to 2018 (Supplementary eTable S5). Although the annual prevalence of IBS fluctuated rather than followed a linear trend from 2012 to 2018, a significant decrease was observed after multivariate adjustment (0.94 [95% CI 0.94-0.94]; *p* < 0.001). In contrast, increasing age (1.02 [95% CI 1.02-1.02]; *p* < 0.001) and female gender (1.12 [95% CI 1.12-1.12]; *p* < 0.001) were associated with increasing prevalence (Supplementary eTable S7).

### Baseline Characteristics

Among the 1193490 IBS patients, 646875 patients (54.20%) were females. The mean age was 49.94 ± 17.28 years. Most patients received an IBS diagnosis from outpatient clinics (99.74%). 56.66% of IBS patients lived in urbanized areas. As for the insurance premium level, 808560 patients (67.75%) were in the group of >18780 NTD (Table 1).

**TABLE 1:** Demographic characteristics of irritable bowel syndrome population.

Characteristics	Number (%) N = 1,193,490
Age, mean (SD)	49.94 (17.28)
Age group	
20-29	163481 (13.70)
30-39	217746 (18.24)
40-49	215684 (18.07)
50-59	234667 (19.66)
60-69	184573 (15.46)
≥70	177339 (14.86)
Gender	
Male	546615 (45.80)
Female	646875 (54.20)
Urbanization (missing = 39507)	
Urban	653902 (54.79)
Suburban	328792 (14.35)
Rural	171289 (14.35)
Premium (missing = 157734)	
>18780	227196 (19.04)
18780-29000	430742 (36.09)
29000>	377818 (31.66)

Diagnostic procedures, comorbidities, and comedications of IBS population were summarized in Table 2. Abdominal ultrasonography (17.72%) was the most common diagnostic procedure used before IBS diagnosis. The utilizations of abdominal ultrasonography, upper GI endoscopy, and lower GI endoscopy significantly increased after IBS diagnosis. Lower GI endoscopy had the greatest increase (from 6.88 to 22.58%, SMD = 0.45).

**TABLE 2:** Diagnostic procedure, comorbidity, and comedication of irritable bowel syndrome population in Taiwan.

Characteristics	Number (%) N = 1,193,490		SMD
	One year before index date	One year after index date	
Diagnostic procedure			
Upper GI endoscopy	150605 (12.62)	243884 (20.43)	0.21
Enteroscopy	15679 (1.31)	29279 (2.45)	0.08
Lower GI endoscopy	82110 (6.88)	269461 (22.58)	0.45
Ultrasonography	211467 (17.72)	299404 (25.09)	0.18
Abdominal X-ray	48575 (4.07)	69811 (5.85)	0.08
Non-gastrointestinal comorbidity			
Hypertension	286886 (24.04)	299842 (25.12)	0.03
Diabetes	135762 (11.38)	146165 (12.25)	0.03
Dyslipidemia	186833 (15.65)	200341(16.79)	0.03
Chronic kidney diseases	29829 (2.50)	36598 (3.07)	0.03
Chronic liver diseases	105731 (8.86)	132496 (11.10)	0.07
Asthma	38654 (3.24)	40714 (3.41)	0.01
COPD	42233 (3.54)	46109 (3.86)	0.02
Allergic rhinitis	115174 (9.65)	120868 (10.13)	0.02
Chronic fatigue syndrome	20791 (1.74)	22477 (1.88)	0.01
Fibromyalgia	97871 (8.20)	102769 (8.61)	0.01
Migraine	17666 (1.48)	18232 (1.53)	0.00
Obesity	3409 (0.29)	3869 (0.32)	0.01
Overactive bladder	1506 (0.13)	2008 (0.17)	0.01
Gastrointestinal comorbidity			
Biliary events	4060 (0.34)	6022 (0.50)	0.03
Chronic pancreatitis	2453 (0.21)	4049 (0.34)	0.03
Cholelithiasis	20197 (1.69)	27360 (2.29)	0.04
Gastritis and duodenitis	191938 (16.08)	195185 (16.35)	0.01
Gastroenteritis and colitis	123108 (10.31)	105158 (8.81)	0.05
GERD	118580 (9.94)	182976 (15.33)	0.16
Infectious enterocolitis	36955 (3.10)	32892 (2.76)	0.02
Gastrointestinal comorbidity			
Gastric functional diseases	87886 (7.36)	119065 (9.98)	0.09
Intestinal functional diseases	60234 (5.05)	68408 (5.73)	0.03
Peptic ulcer	176261 (14.77)	273381 (22.91)	0.21
Psychiatric comorbidity			
Depression	54753 (4.59)	60939 (5.11)	0.02
Anxiety	112052 (9.39)	139471 (11.69)	0.07
Alzheimer’s disease	1530 (0.13)	2011 (0.17)	0.01
Bipolar	5980 (0.50)	6974 (0.58)	0.01
Dementia	17279 (1.45)	21196 (1.78)	0.03
Parkinson’s disease	9820 (0.82)	11597 (0.97)	0.02
Psychotic disorders	11094 (0.93)	11360 (0.95)	0.00
Sleep disorders	165828 (13.89)	183037 (15.34)	0.04
Stress related disorders	3023 (0.25)	3834 (0.32)	0.01
Somatoform Disorders	17597 (1.47)	19059 (1.60)	0.01
Co-medication			
Simethicone	190354 (15.95)	325259 (27.25)	0.28
Propulsives	246045 (20.62)	362140 (30.34)	0.22
Antacid	333592 (27.95)	424416 (35.56)	0.16
H2-blocker	212761 (17.83)	305026 (25.56)	0.19
Proton pump inhibitors	129533 (10.85)	246910 (20.69)	0.27
Urinary antispasmodics	44419 (3.72)	65504 (5.49)	0.08
Anxiolytics	289200 (24.23)	334614 (28.04)	0.09

Abbreviation: COPD, chronic obstructive pulmonary disease; GERD, gastroesophageal reflux disease; GI, gastrointestinal; SMD, standardized mean difference.

Hypertension (24.04%), dyslipidemia (15.65%), and diabetes (11.38%) were the prevalent non-GI comorbidities within 1 year before index date. These comorbidities remained prevalent after index date. Peptic ulcers (14.77%) and GERD (9.94%) were the common GI comorbidities before index date. The rates of peptic ulcer and GERD significantly increased at 1 year after index date. Sleep disorders (13.89%) and anxiety (9.39%) were the most frequent psychiatric disorders accompanying patients with IBS within 1 year before index date. The rates of sleep disorder and anxiety were increased by more than 1% at 1 year after IBS diagnosis, despite no significant difference.

Antacids (27.95%) were the most common comedications in patients with IBS before index date. The prescribing rate of propulsives, simethicone, antacids, H2-blockers, and proton pump inhibitors significantly increased after IBS diagnosis.

### Prescribing Pattern

During 1 year follow-up, 355310 patients (29.77%) didn’t receive index treatment. However, 600584 patients (50.32%) used one type of medication, and 237596 patients (19.91%) used two or more. The most common choice of medication was antispasmodics (*n* = 611046, 51.20%), followed by laxatives (*n* = 273919, 22.95%), antidiarrheals (*n* = 178064, 14.92%), probiotics (*n* = 21842, 1.83%), and antidepressants (*n* = 12475, 1.05%). The prescribing rate of otilonium (*n* = 153697, 25.15%) was the highest one among the class of antispasmodic. The most frequently prescribed laxative were sennosides (*n* = 115329, 42.10%). Loperamide was the most commonly used antidiarrheal, used in half of the patients who received antidiarrheal (*n* = 94806, 53.24%) (Supplementary eTable S8).

**FIGURE 3:**
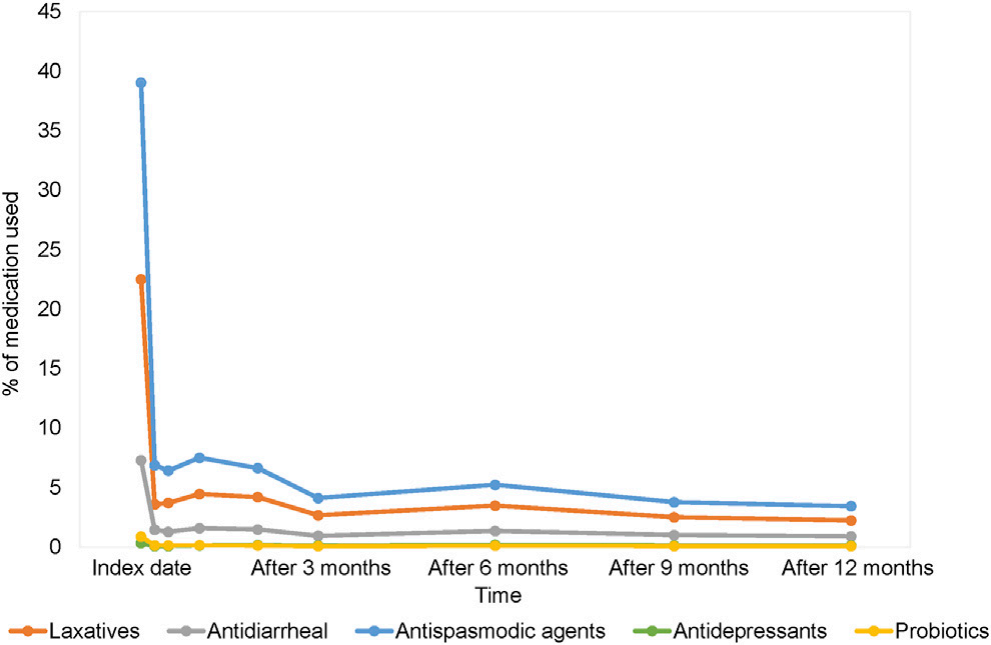
The trend in different pharmacological type of prescription.

Only 48.58% of patients made return visit for IBS after index date. As a consequence, the proportion of healthcare utilization for IBS decreased sharply after index date and showed a significantly decreasing trend (p-value for trend <0.001) (Supplementary eFigure S2). More than 80% of patients didn’t seek medical consultation for IBS in each observation period. Therefore, the prescribing rates of all medications for IBS were decreased considerably after index date (Figure 3; Supplementary Table S9).

## Discussion

The population-based epidemiological study demonstrates the epidemiology, clinical characteristics, and prescribing pattern of IBS patients in Taiwan. The results revealed that the average annual incidence and prevalence of IBS during 2012–2018 in Taiwan were calculated as 106.54 and 181.75 per 10000 population. The incidence and prevalence of IBS increased with advanced age and were prevalent in females. However, a declining trend was observed over time.

In this study, IBS diagnosis was identified by ICD code rather than rigorous research criteria. Therefore, patients may be given other serve diagnoses to avoid refusal of reimbursement (Ellis et al., 2020). The upcoding may frequently occur because of the yearly increase in payment items covered under NHI, leading to the decreasing trend in incidence and prevalence of IBS. Furthermore, clinicians don’t make IBS diagnosis by using the Rome criteria alone in clinical practice (Lacy et al., 2021). The improvement of diagnostic consensus of IBS is also the potential reflection of the decreasing trend (Pan et al., 2016). Moreover, there were two possible explanations of the sharp decrease in prevalence from 2015 to 2016 in this study. First, the diagnostic criteria of IBS were updated to Rome IV in 2016, which eliminated abdominal discomfort from the definition and increased the frequency of abdominal pain (Drossman, 2016). Nevertheless, the frequency of abdominal pain was not so common in Asians (Gwee et al., 2019). Bai et al. (2017) reported that bloating was the main symptom in patients with Rome III-defined IBS in China. 70% of patients experienced bloating, while only 64.6% of patients had recurrent abdominal pain. It might lead to a relatively low incidence of Rome IV defined-IBS. Second, the ICD-9-CM diagnosis code was used for recording diagnosis before 2016, and the ICD-10-CM, which was greater detail than ICD-9-CM, has been used since 2016 in NHIRD (Hsieh et al., 2019). The number of codes available in ICD-10-CM increased nearly 5-fold than ICD-9-CM. However, changes in prevalence of some diseases related to the transition to ICD-10-CM have been observed (Yoon and Chow, 2017). Therefore, the transition may be the potential reason for the decreased prevalence and incidence of IBS despite no literature on the impact of the transition in IBS. It is possible that clinicians less enter IBS diagnosis codes for reimbursements because of the expanded diagnosis code. Further studies are needed to be assess this issue in the future.

The prevalence of IBS decreased modestly with increasing age in most studies, despite no significant difference (Black and Ford, 2020). However, the prevalence significantly increased with age in our study. A possible explanation was that older people are more likely to seek healthcare services due to the higher risk of multiple comorbidities. Therefore, the higher incidence in older patients may reflect their frequent healthcare-seeking behaviour (Locke et al., 2004).

The rate of lower GI endoscopy dramatically increased at 1 year after index date. The increasing trend might be attributed to clinical practice and screening policy in Taiwan. Colonoscopy and sigmoidoscopy were recommended for the differential diagnosis between IBS and GI organic diseases, especially in the patients with alarm features (Gwee et al., 2019; Moayyedi et al., 2019; Fukudo et al., 2021; Lacy et al., 2021). Furthermore, colorectal cancer is the commonly diagnosed cancer in Taiwan. National cancer screening program has been implemented to reduce the mortality of colorectal cancer since 2004, which are subsidized for citizens aged 50–75 years every 2 years (Wang et al., 2018). Therefore, patients aged 50 to 75 with IBS were more likely referred for colonoscopy.

The prevalent coexist non-GI comorbidities in our study included hypertension, dyslipidemia, and diabetes. These have generally been considered unrelated to IBS. Nonetheless, compared with the general population, metabolic syndrome was more prevalent in patients with IBS. IBS was possibly positively associated with metabolic syndrome due to the shared pathophysiology. The alteration of gut microflora was the potential mechanism between IBS and metabolic syndrome (Guo et al., 2014; Bayrak, 2020). Imbalance gut microbiota may cause immune activation, leading to disruption of the gut barrier. The translocation of microbial products, such as endotoxin and tumour necrosis factor-alpha (TNF-α), occurs from the GI tract to systemic sites and results in systemic chronic inflammation and organ damage (Wang et al., 2020). It has been considered as a trigger for the development of metabolic syndrome. Moreover, alteration of gut microbiota was also associated with the development of hepatic injury (Albillos et al., 2020). Lee et al. (2016) suggested that patients with IBS had significantly higher prevalence of metabolic syndrome and elevated ALT and γ-GT levels. Thus, IBS with alteration of gut microbiota is possibly related to metabolism syndrome and liver disease through the same pathophysiology. However, the evidence of the relationship between IBS and metabolism syndrome as well as IBS and liver diseases is limited. Further studies are needed to verify the hypothesis.

In our study, the prevalence of GERD and peptic ulcers significantly increased after IBS diagnosis. GERD was reported to be related to IBS. The overlap with GERD in patients with IBS has been widely reported, ranged from 3 to 79% (de Bortoli et al., 2018). Compared with non-IBS patients, patients with IBS had significantly increased risks for developing GERD (Whitehead et al., 2007; Yarandi et al., 2010; Faresjö et al., 2013). Moreover, patients with GERD also had a higher risk of IBS (Ruigómez et al., 2009). Abnormal GI motility, visceral hypersensitivity, and neural mechanisms could be unified mechanisms between IBS and GERD (de Bortoli et al., 2018). However, there is currently no evidence to support the association between IBS and peptic ulcers. Therefore, the increasing prevalence of peptic ulcers among patients with IBS may result from the frequent use of diagnostic procedures after IBS diagnosis (Whitehead et al., 2007). Besides, further studies are needed to be carried out on the relevance between IBS and peptic ulcers.

IBS has been considered a gut-brain disorder due to the high association between psychological conditions and IBS (Masuy et al., 2020). Patients with either IBS or psychiatric disorders had a significantly higher risk of developing the other condition. In a meta-analysis, patients with either anxiety or depression had a twofold risk of developing IBS during 3 months to 8 years follow-up time (Sibelli et al., 2016). In addition, Zamani et al. demonstrated that the odds of developing anxiety and depression in IBS were three times greater than in non-IBS patients (Zamani et al., 2019). Nonetheless, the prevalence of anxiety and depression in our study did not significantly increase after IBS diagnosis. The main reason was the relatively short follow-up time that was insufficient to detect the onset of both disorders.

Propulsives, simethicone, antacids, H2-blockers, and proton pump inhibitors were prescribed more frequently to patients at 1 year after IBS diagnosis. The primary explanation was the increased prevalence of GERD and peptic ulcers, related to shared pathophysiology with IBS and increased diagnostic investigation after IBS diagnosis. Moreover, the clinical practice in IBS management may contribute to the increasing prescribing rate of propulsives and simethicone. Mosapride, a prokinetic 5-HT4 receptor agonist available in Taiwan, is recommended for patients with IBS-C in Japanese IBS management guidelines (Fukudo et al., 2021). Therefore, mosapride may be used to relieve symptoms of IBS in Taiwanese clinical practice. Moreover, the combination of alverine and simethicone had a greater effect on improving abdominal pain and discomfort than the usual treatment in patients with IBS (Ducrotte et al., 2014). Simethicone might be prescribed to IBS patients as add-on therapy in Taiwan. Additionally, the supplemental use of high-dose simethicone before colonoscopy had a significant effect on improving the quality of bowel preparation. As a consequence, oral simethicone was recommended for bowel preparation in the European Society of GI Endoscopy Guideline (Hassan et al., 2019). The higher rate of colonoscopy utilization in our study might also contribute to the increased rate of simethicone use.

Despite scarce literature on prescribing patterns of IBS, there was a great variety between studies. An England study showed that 23.61% of patients didn’t receive any treatment, and 67.18, 14.33, and 2.07% of patients used antispasmodics, laxatives, and antidiarrheals at the first week after IBS diagnosis (Ruigómez et al., 1999). Rangan et al. (2020) demonstrate that linaclotide and lubiprostone were prescribed to 43.1 and 32.1% of patients with IBS-C; antispasmodics and diphenoxylate were prescribed to 51.0 and 33.5% of patients with IBS-D. Sayuk et al. (2017) reported that 34 and 16% of IBS-D patients used probiotics and antidepressants to manage symptoms of IBS in America. The variations might be affected by different features of IBS, availability of efficacious medications, and clinical practices between countries. In our study, 29.77% of patients with IBS did not receive index treatment within 1 year after diagnosis. The high proportion of non-users was attributed to two reasons. First, pharmacologic agents would not be prescribed for patients with symptoms that didn’t impair quality of life. Second, over-the-counter (OTC) medication was a treatment option for the management of IBS. Fibre, PEG, and peppermint oil have been confirmed to improve IBS symptoms and recommended for IBS management in several guidelines (Gwee et al., 2019; Moayyedi et al., 2019; Fukudo et al., 2021; Lacy et al., 2021). Nevertheless, most OTC medications are not reimbursed by NHI, leading to OTC medications records not being obtained in NHIRD.

Antispasmodics were the most commonly used medication type for IBS, followed by laxatives and antidiarrheals. The use of these medications might reflect a certain extent of clinical symptoms in IBS patients who sought healthcare service in Taiwan. The prescribing rates of antidepressants and probiotics were low in Taiwan. Antidepressants are off-label prescriptions for IBS treatment, and therefore seldom prescribed to IBS patients without psychiatric disorders. As mention above, most OTC medications, including probiotics, are not covered in NHI, resulting in the low prescribing rates of probiotics in our study.

In Taiwan, several efficacious drugs for overall symptoms improvement in IBS patients, such as linaclotide and lubiprostone, are unavailable. Consequently, the medications whose effects on IBS symptoms relief are currently unclear frequently used for IBS management in Taiwan. For example, sennoside and magnesium oxide were commonly prescribed to IBS patients. Although both of them improve consistency and frequency of bowel movements, their effectiveness in improving abdominal pain and quality of life in IBS patients remains unclear (Fukudo et al., 2021). Moreover, the effectiveness of most antidiarrheals available in Taiwan is also unclear despite the frequency of use. Therefore, further studies are needed to support their efficacy in improving the individual IBS symptoms. Furthermore, the Taiwan government needs to deliberate the introduction of other efficacious medications for IBS.

The treatment duration for IBS should be tailored to patients based on the severity of symptom severity. For example, an entire treatment for IBS is unnecessary in patients with mild symptoms for one or 2 days, whereas patients with symptoms for a couple of weeks need a long-term treatment course (Evangelista, 2012). In our study, the proportions of healthcare utilization and medication used for IBS sharply decreased after IBS diagnosis. It may reflect that patients with IBS have a short symptom duration or receive a short course of treatment for IBS in Taiwan. Furthermore, several studies have been suggested that some patients were dissatisfied with their pharmacological treatment. Rangan et al. (2020) revealed that less than 20% of patients were very satisfied with their treatment, except for medications approved by the US Food and Drug Administration (FDA) for IBS. Sayuk et al. (2017) reported that only 20% of IBS-D patients were satisfied with the overall treatment. Therefore, patients dissatisfied with conventional treatments would seek other forms of treatment that might lead to a low return visit rate after IBS diagnosis (Li and Li, 2015).

To the best of our knowledge, this study is the first population-based study investigating the prescribing pattern of IBS in Taiwan. All IBS population in our study was obtained from the NHIRD, which aggregates comprehensive medical claim data for over 99% of Taiwanese population. Thus, we can provide the whole picture of epidemiology and prescribing pattern of IBS in Taiwanese population and avoid the selection and recall bias. However, there were still some limitations in our study. First, in order to evaluate the broadness of epidemiology and prescribing pattern of IBS, we didn’t exclude the organic diseases that mimic symptoms of IBS, such as coeliac disease and inflammatory bowel disease. Although miscoding may become a problem, the total number of these organic diseases in our population was low (Supplemetary eTable S10). Therefore, this is not sufficient to impact our results. Second, this study reflects the health care utilization for IBS rather than the true incidence and prevlaence of IBS. In Taiwan, 53.02% of patients with IBS did not seek healthcare for their symptoms (Lu et al., 2003). In addition, patients may be given other diagnoses to more severe ones rather than IBS for reimbursements (Hsieh et al., 2019). Therefore, the incidence and prevalence based on claim data might be underestimated. Moreover, not all OTC medications are reimbursed by NHI. For example, prebiotics and synbiotics and most probiotics are out-of-pocket costs for medical care in NHI. The proportion of medications used for IBS might be underestimated. Third, the disease severity and subtype of IBS are unavailable in NHIRD. It is difficult to provide epidemiological and other information on different disease severity and subtype of IBS in our study. Furthermore, it is challenging to evaluate treatment satisfaction and the reasons for treatment termination. Hence, more studies are needed to investigate further pharmacoepidemiological of IBS.

## Conclusion

In conclusion, the incidence and prevalence of IBS gradually decreased from 2012 to 2018. Although they might be underestimated, this study still provided a lower bound estimate. Antispasmodics were the most frequently prescribed for IBS, followed by laxatives and antidiarrheals. However, the proportion of healthcare utilization for IBS and the prescribing rates of all medications decreased sharply after IBS diagnosis. The findings of this study provided the whole picture of the epidemiology and prescribing pattern of the IBS population in Taiwan, which should be useful for establishing clinical practice guidelines and economic modeling and informing health policy decisions for IBS.

## Data Availability

The corresponding author (C-YC) had full access to all the data in the study and takes responsibility for the integrity of the data and the accuracy of the data analysis. Data are available from the National Health Insurance Research Database (NHIRD) published by the Bureau of National Health Insurance (BNHI) of the Ministry of Health and Welfare. The conclusions presented in this study are those of the authors and do not necessarily reflect the views of the BNHI, the Ministry of Health and Welfare. Requests to access the datasets should be directed to jk2975525@hotmail.com.
