# Supplementary material for: Epidemiology, Clinical Features, and Prescribing Patterns of Irritable Bowel Syndrome in Taiwan
Source: Front Pharmacol. 2021 Dec 9;12:788795. doi: 10.3389/fphar.2021.788795 (PMC8717931; doi:10.3389/fphar.2021.788795)

Supplemental Online Content

Epidemiology, Clinical Features, and Prescribing Patterns of Irritable Bowel Syndrome in Taiwan

eTable 1. NHI code for procedure

eTable 2. ICD code for comorbidities

eTable 3. ATC code for comedications

eTable 4. ATC code for prescribing patterns of IBS

eTable 5. Incidence and prevalence of IBS in Taiwan from 2012 to 2018

eTable 6. Multivariate Poisson regression model on incidence of irritable bowel syndrome in Taiwan from 2012-2018

eTable 7. Multivariate Poisson regression model on prevalence of irritable bowel syndrome in Taiwan from 2012-2018

eTable 8. The choice of treatment among irritable bowel syndrome patients within one year after index date

eTable 9. Prescription pattern of IBS patients within 1 year after index date

eTable 10. The number of organic diseases that mimic symptoms of IBS

eFigure 1. Flowchart of study population

eFigure 2. Trend in healthcare utilization for irritable bowel syndrome

eTable 1. NHI code for procedure

Procedure	NHI code
GI Endoscopy	
Upper GI endoscopy	28010B, 28013C, 28017C, 49014C, 49016C, 49023C, 49025C, 49026C, 49027C
Lower GI endoscopy	28015C, 28016C, 47043B, 47043C, 47083C
Ultrasound	
Abdominal ultrasound	19001C, 19005B1, 19009C
X-ray	
GI series	33006B, 32007C, 32008C, 33008B, 33009B, 33010B, 33011B

Abbreviation: GI, gastrointestinal; NHI, National Health Insurance

eTable 2. ICD code for comorbidities

Comorbidity	ICD-9-CM	ICD-10-CM
Non-GI diseases		
Hypertension	401-405	I10-I15
Hyperlipidemia	272	E75, E77, E78
Diabetes	250	E10-E14
Chronic kidney diseases	583, 585, 586, 587	N05-N07,N16, N18
Chronic liver disease	571-573, 0702, 0703, 07041, 07044, 07051, 07054 V427, Z944	K70, K71, K73-K75, K721, K729, K760, K762-K769, K77, B16, B171, B180-B182, B191, B192
Asthma	493	J45
COPD	491, 492, 496, 4932	J41, J42, J44
Allergic rhinitis	477	J30
Chronic fatigue syndrome	7807,	R53, G933
Fibromyalgia	7291	M608, M609, M791, M797
Migraine	346	G43
Obesity	278.0, 278.01, 278.1	E65, E66
Overactive bladder	59651	N3281
GI disease		
Biliary event	576	K83
Chronic pancreatitis	5771, 5772, 5778, 5779	K86
Cholelithiasis	574	K80
Gastritis and duodenitis	535	K29
Gastroenteritis and colitis	558	K52
GERD	53011, 53081	K21
Infectious enterocolitis	001–009, 11285	A00-A09, B37.82
Gastric functional disease	5368, 5369	K30, K3189, K319
Intestinal functional disease	5648, 5649	K592, K598, K599
Peptic ulcer	531-534	K25-K28

eTable 2. ICD code for comorbidities (continued)

Comorbidity	ICD-9-CM	ICD-10-CM
Psychiatric disorders		
Depression	2962–2963, 3004, 311, 3090, 3091	F32, F33, F4321
Anxiety	29384, 3000, 3002, 3002, 30921	F064, F40, F41, F930
Alzheimer’s disease	3310	G30
Bipolar disorder	2964, 2965, 2966 , 2967, 2968	F31
Dementia	290, 294,	F01, F02, F03
Parkinson’s disease	332	G20, G21
Psychotic disorders	295, 297, 298	F20, F22, F23, F24, F28, F29
Sleep disorders	3074, 7805	F51, G47
Stress related disorders	308, 3098	F430, F431, F438
Somatoform Disorders	3001, 3007,3008, 306, 3078	F44,F45

Abbreviation: COPD, chronic Obstructive Pulmonary Disease; GERD, gastroesophageal reflux disease; GI, gastrointestinal;

eTable 3. ATC code for comedications

Class	ATC code
Simethicone	A03AX13, A02AF02
Propulsives	A03FA01, A03FA03, A03FA04, A03FA09
Antacids	A02AA02, A02AA04, A02AB, A02AB01, A02AB03, A02AB04, A02AB91, A02AB92, A02AB93, A02AB94, A02AC01, A02AD01, A02AD02, A02AD03, A02AD04, A02AF01, A02AF02, A02AG, A02AH, A02AX
Histamine-2-receptor antagonist	A02BA01, A02BA02, A02BA03, A02BA04, A02BA06
Proton pump inhibitor	A02BC01, A02BC02, A02BC03, A02BC04, A02BC05, A02BC06
Urinary antispasmodics	G04BD, G04BD02, G04BD04, G04BD06, G04BD07, G04BD08, G04BD09, G04BD12
Anxiolytic	N05BA01, N05BA02, N05BA03, N05BA04, N05BA05, N05BA06, N05BA08, N05BA09, N05BA11, N05BA12, N05BA16, N05BA17, N05BA22, N05BA91, N05BB01, N05BC01, N05BE01, N05BX01

eTable 4.ATC code for prescribing patterns of IBS

Class	Medication	ATC code
Laxative	Bisacodyl, Cascara, Castor oil, Ispaghula, Frangula, Lactitol, Lactulose, Magnesium carbonate, Magnesium hydroxide, Magnesium oxide, Polyethylene glycol, Scutellariae, Sodium phosphate, Sterculia	A06AB02, A06AB20, A06AB07, A06AB05, A06AC01, A06AC51, A06AD12, A06AD11, A06AD10, A02AD01, A02AF02, A02AD04, A02AD01, A02AA04, A06AD02, A02AA02, A02AF02, A02AD01, A06AC08, A06AD15, A06AB06, A06AB20, A06AD17, A06AC03, A06AC53
Antidiarrheal	Albumin tannate, Bismuth, Charcoal, Diosmectite, Kaolin, Loperamide, Pectin, Methylenethymoltannin, Racecadotril, Cholestyramin, Colestipol, Colextran	A07XA01, A02BX05, A02BX91, A02BX12, A07XA51, A07BA01,A07BC05, A07BC30, A07XA51, A07DA03, A07BC30, A07XA04, C10AC01, C10AC02, C10AC03
Antispasmodic	Alverine, Atropine, Belladonna, Benactyzine, Butropium, Camylofin, Clidinium, Cyclonium, Dicyclomine, Ethaverine, Fenoverine, Flopropione, Glycopyrrolate, Homatropine, Hyoscyamine, Mebeverine, Mepenzolate, Methylscopolamine, Otilonium, Oxyphencyclimine, Papaverine, Pinaverium, Piperidolate, Pipethanate, Propantheline, Scopolamine, Timepidium, Trimebutine, Trospium, Valethamate	A03AX08, A03BB02, A03BA01, A03CB02, A03AB91, A03AA03, A03CA02, A03AB92, A03AA07, A03AD91, A03AX05, A03AX95, A03AB02, A03BB06, A03BA03, A03CB31, A03AA04, A06AC51, A03AB12, A03BB03, A03AB06, A03AA01, A03AD01, A03AX04, A03AA30, A03AA30, A03BA92, A03AB05, A03BB01, A03AB19, A03CA01, A03AA05, A03AB20, A03AB93
Antidepressant	Amitriptyline, Citaloprim, Clomipramine, Dothiepin, Doxepin, Escitalopram, Fluoxetine, Fluvoxamine, Imipramine, Maprotiline, Paroextine, Sertraline	N06AA02, N06AA09, N06CA01, N06AB04, N06AA04,N06AA16, N06AA12, N06AB10, N06AB03, N06AB08, N06AA21, N06AB05, N06AB06
Probiotic	-	AF07

eTable 5. Incidence and prevalence of IBS in Taiwan from 2012 to 2018

	2012	2013	2014	2015	2016	2017	2018	Change (%)
**Incidence per 10 000 population**								
**Total (crude)**	134.79	116.85	113.92	105.77	91.13	93.99	89.35	-33.71
**Female**	146.48	125.62	122.98	113.34	96.92	98.30	93.36	-36.26
**Male**	122.86	107.47	104.67	98.04	85.19	89.56	85.23	-30.63
**Total (Adjusted)**	134.79	116.44	113.31	105.08	90.37	93.21	88.37	-34.34
**Female**	146.48	125.13	122.28	112.56	96.08	97.30	92.11	-37.12
**Male**	122.86	107.13	104.24	97.58	84.68	89.13	84.62	-31.12
**Prevalence per 10 000 population**								
**Total (crude)**	192.46	187.11	191.46	188.43	167.94	175.34	169.47	-11.94
**Female**	206.98	199.81	204.55	200.33	177.38	183.33	177.18	-14.40
**Male**	177.66	174.12	178.04	176.19	158.22	167.08	161.49	-9.10
**Total (Adjusted)**	192.46	185.96	189.52	186.05	165.47	172.65	166.38	-13.55
**Female**	206.98	198.49	202.29	197.52	174.45	179.92	173.28	-16.28
**Male**	177.66	173.22	176.58	174.51	156.49	165.37	159.43	-10.26

Change (%): percentage of changes in the incidence of IBS between 2003 and 2013

eTable 6. Multivariate Poisson regression model on incidence of irritable bowel syndrome in Taiwan from 2012-2018

Variable	Crude IRR	95% CI	P value	Adjusted IRR	95% CI	P value
**Calendar year**	0.92	0.92-0.92	<.001	0.91	0.91-0.91	<.001
**Age**	1.01	1.01-1.01	<.001	1.01	1.01-1.01	<.001
**Gender**						
**Male**	-	-		-	-	
**Female**	1.15	1.15-1.16	<.001	1.14	1.14-1.15	<.001
**Urbanization**						
**Rural**	-	-		-	-	
**Suburban**	0.94	0.94-0.95	<.001	0.96	0.95-0.97	<.001
**Urban**	0.99	0.98-0.99	<.001	1.00	1.00-1.01	<.001
**Insurance premium**						
**<18,700**	-	-		-	-	
**18,700-29,000**	1.07	1.07-1.08	<.001	1.13	1.13-1.14	<.001
**>29,000**	1.13	1.12-1.13	<.001	1.20	1.19-1.21	<.001

Abbreviation: CI, confidence interval; IRR, incidence rate ratio

eTable 7. Multivariate Poisson regression model on prevalence of irritable bowel syndrome in Taiwan from 2012-2018

Variable	Crude IRR	95% CI	P value	Adjusted IRR	95% CI	P value
**Calendar year**	0.96	0.96-0.96	<.001	0.94	0.94-0.94	<.001
**Age**	1.02	1.02-1.02	<.001	1.02	1.02-1.02	<.001
**Gender**						
**Male**	-	-		-	-	
**Female**	1.13	1.13-1.14	<.001	1.12	1.12-1.12	<.001
**Urbanization**						
**Rural**	-	-		-	-	
**Suburban**	0.91	0.91-0.92	<.001	0.95	0.95-0.95	<.001
**Urban**	0.93	0.92-0.93	<.001	0.98	0.98-0.98	<.001
**Insurance premium**						
**<18,700**	-	-		-	-	
**18,700-29,000**	1.06	1.06-1.07	<.001	1.15	1.14-1.15	<.001
**>29,000**	1.08	1.08-1.09	<.001	1.21	1.21-1.22	<.001

Abbreviation: CI, confidence interval; IRR, incidence rate ratio

eTable 8. The choice of treatment among irritable bowel syndrome patients within one year after index date

Medication	Number (%)
**Anticonstipation**	**273919 (22.95)**[a]
Bisacodyl	54856 (20.03)
Frangula	44950 (16.41)
Ispaghula	14614 (5.34)
Lactulose	8438 (3.08)
Magnesium carbonate	20422 (7.46)
Magnesium hydroxide	11311 (4.13)
Magnesium oxide	113893 (41.58)
Polyethylene glycol	4591 (1.68)
Sennoside	115329 (42.10)
Sodium phosphate	6752 (2.46)
Sterculia	45162 (16.49)
Other	449 (0.16)
**Antidiarrheal**	**178064 (14.92)**[a]
Bismuth	69802 (39.20)
Cholestyramine	858 (0.48)
Diosmectite	33352 (18.73)
Kaolin	41204 (23.14)
Loperamide	94806 (53.24)
Racecadotril	3 (0.002)
Other	10088 (5.67)
**Antispasmodic agents**	**611046 (51.20)**[a]
Alverine	45402 (7.43)
Atropine	1548 (0.25)
Butropium	7256 (1.19)
Clidinium	18927 (3.10)
Dicyclomine	32517 (5.32)
Flopropione	3415 (0.56)
Homatropine	14336 (2.35)
Hyoscyamine	43486 (7.12)
mebeverine	145211 (23.76)
Mepenzolate	73427 (12.02)
Methylscopolamine	10310 (1.69)
Otilonium	153697 (25.15)

eTable 8. The choice of treatment among irritable bowel syndrome patients within one year after index date (continued)

Medication	Number (%)
**Antispasmodic agents**	**611046 (51.20)**[a]
Pinaverium	97138 (15.90)
Pipethanate	20074 (3.29)
Scopolamine	74783 (12.24)
Timepidium	1932 (0.32)
Trimebutine	9106 (1.49)
Other	5844 (0.96)
**Antidepressants**	**12475 (1.05)**[a]
Amitriptyline	1189 (9.53)
Citaloprim	627 (5.03)
Escitalopram	2971 (23.82)
Fluoxetine	1297 (10.40)
Imipramine	3956 (31.71)
Paroextine	1048 (8.40)
Sertraline	1708 (13.69)
Other	573 (4.59)
**Probiotics**	**21842 (1.83)**[a]

[a] Among all irritable bowel syndrome patients in this study (N=1193490)

eTable 9. Prescription pattern of IBS patients within 1 year after index date

		After index date								
	**Index date N=1193490**	**1 week N=1193185**	**2 weeks N=1192850**	**1 month N=1191976**	**2 months N=1190153**	**3 months N=1188428**	**6 months N=1183869**	**9 months N=1179763**	**12 months N=1175788**	**P for trend**
**Laxatives**	224804 (18.84)	32313 (2.71)	28705 (2.41)	34452 (2.89)	33566 (2.82)	23765 (2.00)	36453 (3.08)	27514 (2.33)	24476 (2.08)	<.001
**Antidiarrheal**	145332 (12.18)	16913 (1.42)	12383 (1.04)	14737 (1.24)	14564 (1.22)	10611 (0.89)	17574 (1.48)	13831 (1.17)	12732 (1.08)	<.001
**Antispasmodics**	559895 (46.91)	78643 (6.59)	59132 (4.96)	67588 (5.67)	62916 (5.29)	43505(3.66)	64612 (5.46)	48984 (4.15)	44553 (3.79)	<.001
**Antidepressants**	8343 (0.7)	1833 (0.15)	1949 (0.16)	2928 (0.25)	3366 (0.28)	2577 (0.22)	3380 (0.29)	2643 (0.22)	2376 (0.20)	<.001
**Probiotics**	18442 (1.55)	2024 (0.17)	1527 (0.13)	1793 (0.15)	1705 (0.14)	1245 (0.10)	1955 (0.17)	1473 (0.12)	1373 (0.12)	<.001

eTable 10. The number of organic diseases that mimic symptoms of IBS (N= 1193490)

	ICD-9-CM	ICD-10-CM	Number	%
**Celiac disease**	5790	K900	22	0.00%
**Inflammatory bowel disease**	555, 556	K50, K51	7257	0.61%
**Colon cancer**	153	C18, C7A02	5992	0.50%
**Rectal cancer**	154	C19, C20, C21, C7A026	3870	0.32%

eFigure 1. Flowchart of study population

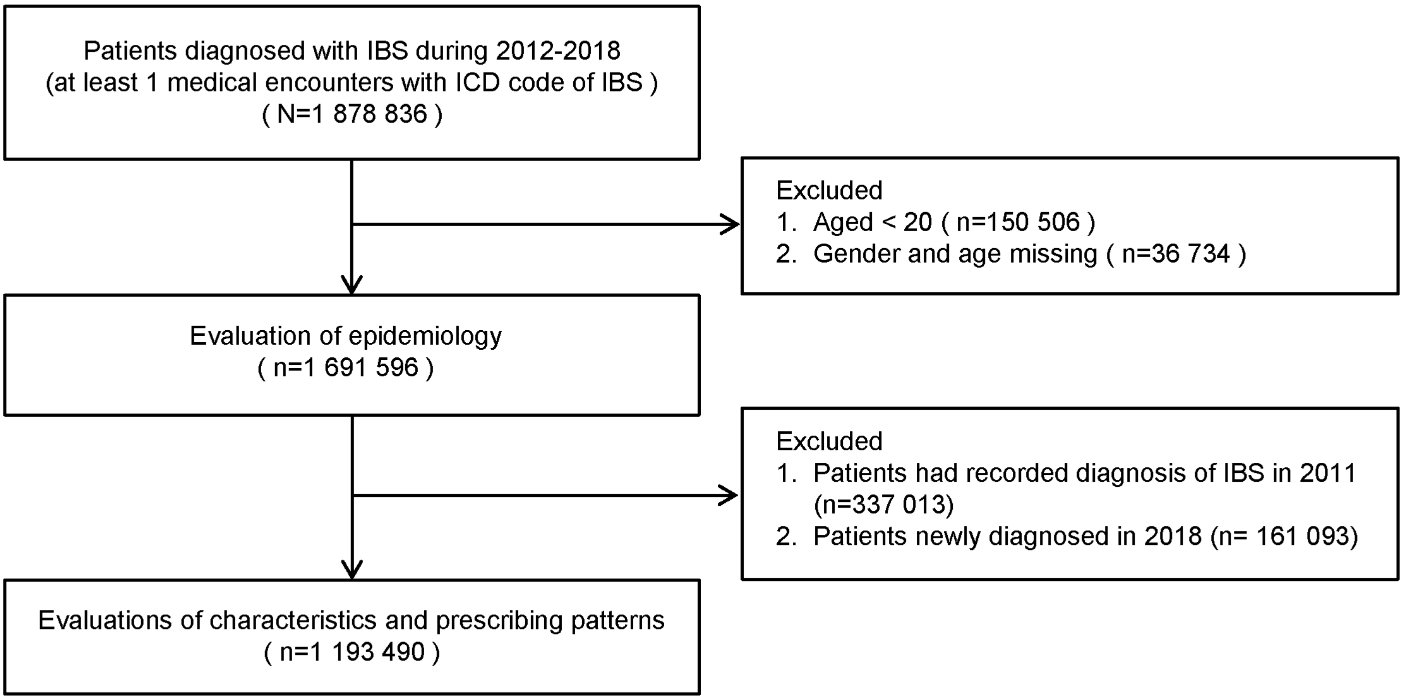

eFigure 2. Trend in healthcare utilization for irritable bowel syndrome

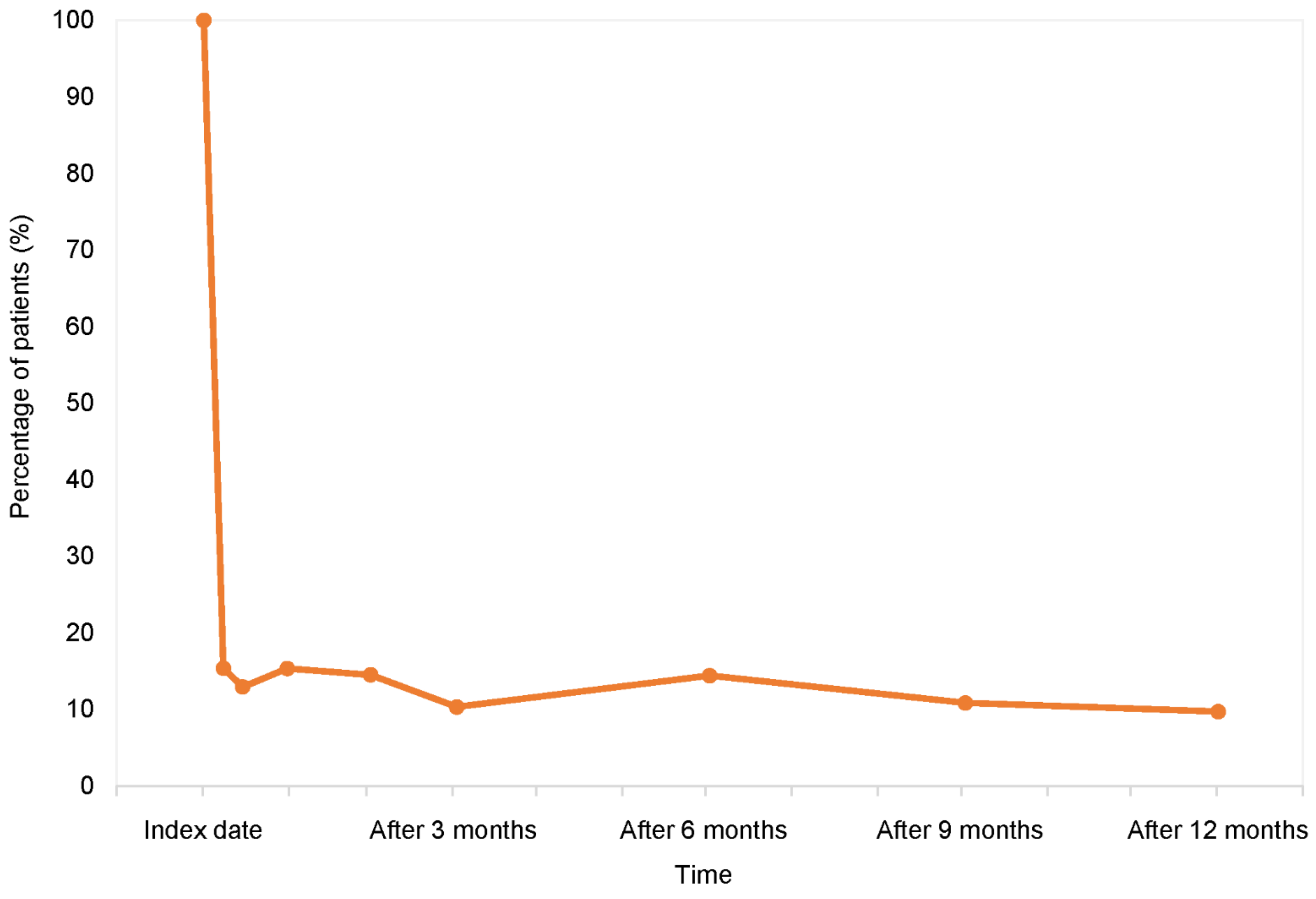

Supplement: Supplementary file 1 [file DataSheet1.PDF]
